# Ulnar-Mammary Syndrome: Clinical Presentation, Genetic Underpinnings, Diagnosis, and Treatment

**Published:** 2014-09-27

**Authors:** Jameson Loyal, Donald R. Laub

**Affiliations:** ^a^University of Vermont College of Medicine; ^b^Fletcher Allen Health Care, Burlington, Vt

**Keywords:** ulnar-mammary syndrome, Schinzel syndrome, genetics, T-box genes, upper extremity anomaly

## DESCRIPTION

CS presented shortly after birth with absent left third, fourth, and fifth fingers, a flexed left elbow with an antecubital pterygium, and hypoplastic inverted, widely spaced nipples. Her right fifth finger was without active or passive flexion. A radiograph showed an absent left ulna.

## QUESTIONS

1. **What is the clinical presentation of a patient with ulnar-mammary syndrome?**
2. **What are the genetic manifestations of ulnar-mammary syndrome?**
3. **What are some important signs for clinicians to detect ulnar-mammary syndrome in patients?**
4. **What types of medical therapies are available for patients with ulnar-mammary syndrome?**

## DISCUSSION

**Figure 1:**
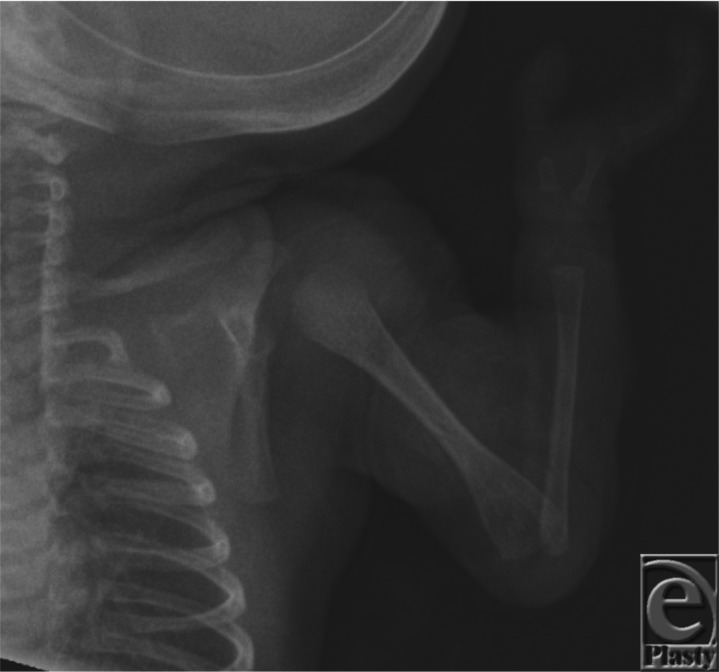
Radiograph showing absence of the ulna and ulnar three digits.

**Figure 2:**
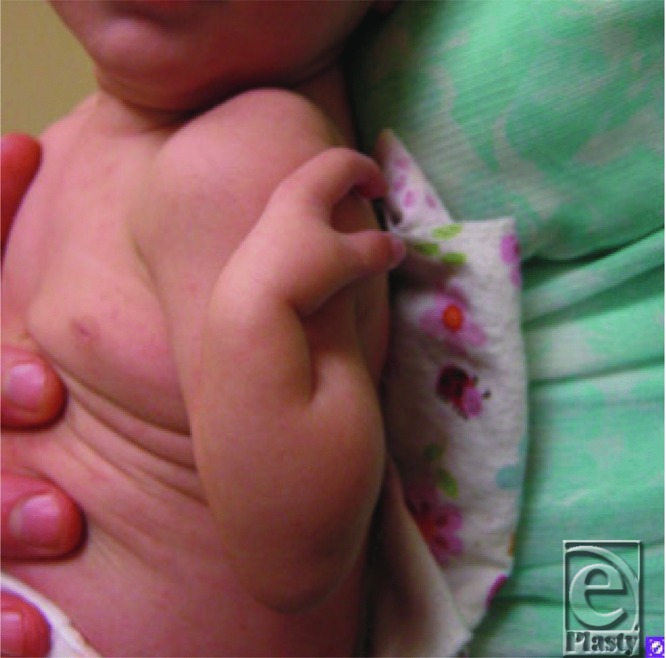
Left Hand, showing only the radial two digits are present.

**Figure 3:**
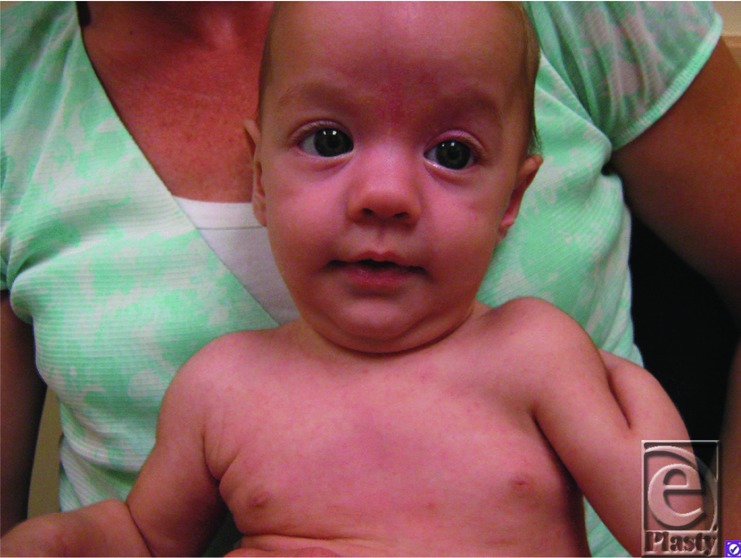
Facial appearance of the patient, demonstrating wide forehead, wide nose and protruding chin.

Ulnar-mammary syndrome (UMS) was first described in 1975 by McKusick.^1^ UMS presents with high variability, and typically with asymmetric presentation. Defects of the postaxial components of the upper extremities may include hypoplastic or missing ulna (Fig 1), camptodactyly, postaxial polydactyly, or missing digits (Fig 2).^2^ Generally, there is hypoplasia of the apocrine glands, mammary glands, the areola, and inverted nipples.^3^ Reduced or inability to lactate and uterine defects can be seen in females.^1^^,^^2^^,^^4^ Delayed puberty and genital hypoplasia is frequently seen in males, resulting in micropenis, cryptorchidism, and/or a shawl scrotum.^4^ Other abnormalities include defects of the heart, endocrine system, teeth, and palate.^3^^,^^4^ Interestingly, malformations of the lower extremities are not reported.^4^ Typically, patients have a wide face, nasal base and tip, and a protruding chin (Fig 3).^5^

UMS is rare; approximately 117 cases have been reported.^4^ UMS is an autosomal dominant condition that exhibits incomplete penetrance. The T-box genes, specifically T-box transcription factor 3 (TBX3) on chromosome 12q23-24.1 are believed responsible for this syndrome.^6^ T-box genes assist in body patterning during embryogenesis serving to map the extremities.^4^ TBX3 is expressed in the upper limb; mammary tissue^3^; placenta; bladder; uterus; liver; heart; and adrenal, pituitary, and thyroid glands.^3^^,^^4^ UMS manifests in patients lacking TBX3 that results from haploinsufficiency, having one functional copy of the TBX3 gene. Haploinsufficiency of transcription factor TBX3 reportedly results from either a deletion or a splice-site error. It is thought that TBX3 deficiencies reduces DNA binding to the transcription factor and contributes to the loss of TBX3's functionality in organ and limb development.^2^^,^^6^ Different mutations within and outside the TBX3 gene demonstrate the phenotypic variability of this disorder. It has been suggested that the organ systems more affected in UMS patients may require different amounts of TBX3 or that other genes in the T-box family might compensate for the lack of TBX3 in other tissues.^7^

Aside from the classical anatomical malformations associated with UMS, physicians should be open to a diagnosis of UMS even when there are subtler physical examination findings. Defects involving the mammary glands or genitalia might not present until after puberty, adding to the complexity of diagnosis.^4^ Meticulous examination of a patient's face for typical UMS findings may help in the diagnosis.^5^ Because of the variability in the severity of UMS seen within families, a UMS diagnosis should not be dismissed even if not previously recognized in a family member. Finally, genetic analysis of TBX3 genes can establish a diagnosis of UMS, especially when physical examination findings are within normal range. UMS patients have defects in the postaxial limbs; patients with preaxial malformations should be examined for the possibility of Holt-Oram syndrome.^2^ As reports identify patients with UMS with cardiac abnormalities, cardiac screening is recommended. In addition, TBX3 may be involved in the development of the hypothalamic-pituitary axis, so brain imaging and hormone testing should be included in the workup of a patient.^3^

Treatment depends on the severity of the patient's condition and can include the following: splinting or surgery to improve elbow range of motion, surgical reconstruction to improve finger function, or excision of supernumerary digits.^4^ Hormonal deficiencies can benefit from hormone replacement therapy.^8^

UMS is a disorder with symptomatology that varies in severity and organ system involvement. Physicians must be cognizant of this disorder even if a patient does not exhibit the severe, classical presentation.
